# Longitudinal High-Resolution Imaging of Retinal Sequelae of a Choroidal Nevus

**DOI:** 10.3390/diagnostics15151904

**Published:** 2025-07-29

**Authors:** Kaitlyn A. Sapoznik, Stephen A. Burns, Todd D. Peabody, Lucie Sawides, Brittany R. Walker, Thomas J. Gast

**Affiliations:** 1 School of Optometry, Indiana University, Bloomington, IN 47405, USA; 2 College of Optometry, University of Houston, Houston, TX 77204, USA; 3 Instituto de Óptica, Consejo Superior de Investigaciones Científicas, 28006 Madrid, Spain

**Keywords:** adaptive optics, choroidal nevus, longitudinal imaging

## Abstract

**Background**: Choroidal nevi are common, benign tumors. These tumors rarely cause adverse retinal sequalae, but when they do, they can lead to disruption of the outer retina and vision loss. In this paper, we used high-resolution retinal imaging modalities, optical coherence tomography (OCT) and adaptive optics scanning laser ophthalmoscopy (AOSLO), to longitudinally monitor retinal sequelae of a submacular choroidal nevus. **Methods**: A 31-year-old female with a high-risk choroidal nevus resulting in subretinal fluid (SRF) and a 30-year-old control subject were longitudinally imaged with AOSLO and OCT in this study over 18 and 22 months. Regions of interest (ROI) including the macular region (where SRF was present) and the site of laser photocoagulation were imaged repeatedly over time. The depth of SRF in a discrete ROI was quantified with OCT and AOSLO images were assessed for visualization of photoreceptors and retinal pigmented epithelium (RPE). Cell-like structures that infiltrated the site of laser photocoagulation were measured and their count was assessed over time. In the control subject, images were assessed for RPE visualization and the presence and stability of cell-like structures. **Results**: We demonstrate that AOSLO can be used to assess cellular-level changes at small ROIs in the retina over time. We show the response of the retina to SRF and laser photocoagulation. We demonstrate that the RPE can be visualized when SRF is present, which does not appear to depend on the height of retinal elevation. We also demonstrate that cell-like structures, presumably immune cells, are present within and adjacent to areas of SRF on both OCT and AOSLO, and that similar cell-like structures infiltrate areas of retinal laser photocoagulation. **Conclusions**: Our study demonstrates that dynamic, cellular-level retinal responses to SRF and laser photocoagulation can be monitored over time with AOSLO in living humans. Many retinal conditions exhibit similar retinal findings and laser photocoagulation is also indicated in numerous retinal conditions. AOSLO imaging may provide future opportunities to better understand the clinical implications of such responses in vivo.

## 1. Introduction

Choroidal nevi are common, benign intraocular tumors present in 4.6–7.9% of the white U.S. population [1]. The prevalence of choroidal nevi is thought to be lower in black, Hispanic, and Asian populations in the U.S. [2]. Globally, the prevalence is unknown due to limited population-based studies but was 2.9% in an adult Chinese population [3] and 1.4% in an adult Malay population [4]. It is estimated that the annual rate of malignant transformation of these lesions is 1 in 8845 and increases with age [1]. Differentiating choroidal nevi from small choroidal melanomas may at times be challenging. Historically, several risk factors for growth of small choroidal melanomas can be summarized by the mnemonic “TFSOM-UHH” or “to find small ocular melanoma using helpful hints” [5,6]. These risk factors include thickness (T) greater than 2 mm, subretinal fluid (F), symptoms (S), orange (O) pigment, margin (M) touching the optic disk, ultrasonographic hollowness (UH), and absence of a halo (H), assessed with fundus examination and b-scan ultrasonography [5,6]. Fundus angiography has also been used for decades to aid in the monitoring of high-risk choroidal nevi to assess blood flow, which may be increased in malignant tumors [7,8]. More recently, advances in posterior segment clinical imaging modalities have aided in the diagnosis and differentiation of a choroidal nevus from a melanoma and in the detection and monitoring of retinal sequalae that may occur. These modalities include optical coherence tomography (OCT), OCT angiography (OCTA), fundus autofluorescence (FAF), and widefield imaging [9,10,11]. With modern imaging techniques incorporated, Shields and colleagues have modified their original mnemonic to “TFSOM-DIM”, which stands for “to find small ocular melanoma doing imaging”, and replaces the “M” with “melanoma hollow” using ultrasound and “DIM” with “diameter > 5 mm” using fundus photography [8]. Other imaging modalities, like OCT and FAF, can be used to detect SRF and orange pigment. When multimodal imaging is not accessible, the more recent “MOLES” mnemonic may also be used to assess the risk of malignant transformation and stands for mushroom shape (M), orange pigment (O), large size (L), enlargement (E), and subretinal fluid (S) [12]. Thus, choroidal nevi with any of these risk factors present should be carefully evaluated and monitored to ensure early detection of malignant transformation.

Choroidal nevi can cause outer retinal changes, likely due to the chronicity of the lesion’s interference with outer retinal physiology, but they are not always indicative of growth or malignant transformation. These retinal sequalae are often overlying the choroidal nevus and include disruption of the retinal pigment epithelium (RPE); thinning, disruption, and/or degeneration of the RPE and/or photoreceptors; drusen formation; upregulation of lipofuscin (seen clinically as orange pigment); and choroidal neovascularization [13]. A breakdown of the outer RPE barrier may occur, leading to subretinal fluid [14]. When these changes are significant and affect the macular region, they have the potential to cause serious vision loss even though the lesion is benign [15,16]. In fact, approximately 50% of vision loss due to choroidal nevi is the result of SRF within the macular region [15] and is often treated with laser photocoagulation [17,18]. In choroidal nevi that do have outer retinal changes, a multimodal imaging approach can be used to monitor such changes. However, these outer retinal changes are not unique to nevi and, instead, may demonstrate a generalized response of the outer retina to physiological stress, as similar changes are observed fundoscopically and with posterior segment imaging modalities in other chronic retinal conditions, including central serous chorioretinopathy (CSC) [19,20,21], polypoidal choroidal vasculopathy [21], and numerous retinal degenerations [22] where the choroid and outer retina are implicated.

While not currently used in clinical assessment, adaptive optics (AO) retinal imaging, which provides cellular- and subcellular-level resolution [23], has been used to investigate photoreceptor alterations due to a choroidal nevus [24] and melanoma [25]. AO imaging is also used to assess outer retinal changes in numerous conditions [19,23,26] and the retinal response to laser treatment [27] and injury [28]. Here, we used adaptive optics scanning laser ophthalmoscopy (AOSLO) and spectral-domain OCT (SD-OCT) to follow retinal changes associated with a high-risk choroidal nevus longitudinally. Specifically, we focused our imaging studies on areas of subretinal fluid and focal laser photocoagulation. Although these findings are associated with a choroidal nevus, our observations during these imaging sessions can be applied to other retinal pathologies with similar findings to improve understanding of the changes that occur in living humans at the cellular level.

## 2. Materials and Methods

### 2.1. Subjects

**Figure 1:**
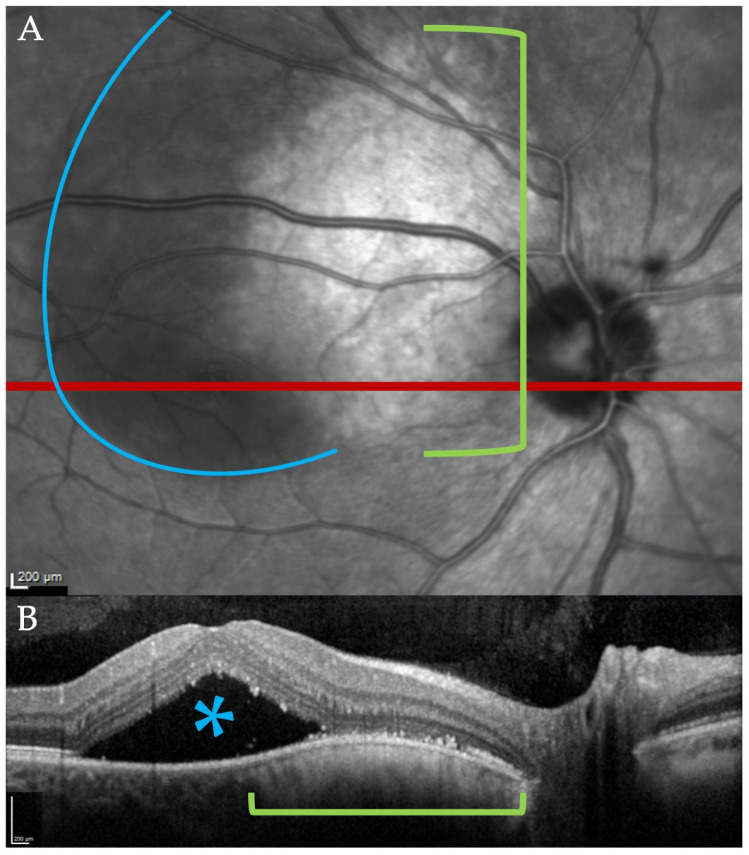
Infrared (IR) and optical coherence tomography (OCT) image of the choroidal nevus and associated subretinal fluid (SRF). (**A**). The choroidal nevus is visualized as a hyperreflective whiteish lesion in the IR image, and the length of the nevus is demonstrated by the green brackets. SRF is located temporal to the nevus and appears dark on the IR image. The edge of the SRF is outlined in blue. (**B**). The OCT b-scan (location corresponds to red line in (**A**)) demonstrates SRF at the macula (blue asterisk), and the hyper-reflective nevus is visible under the RPE in the choroid (green bracket).

This study followed two participants over time. A 31-year-old white female with a high-risk choroidal nevus in the right eye (Figure 1A) was imaged longitudinally in our lab. The participant had an unremarkable medical history and was monitored by our clinic and an external retinal oncology service. The choroidal nevus was approximately 4 mm × 4 mm in size and located adjacent to the superior temporal disk margin and extended into the macula (Figure 1A). There was subretinal fluid (SRF) associated with the nevus (Figure 1) that waxed and waned over the duration of the study. Multimodal imaging was performed in the affected eye in this participant during 21 research visits over 18 months. The patient underwent laser photocoagulation at the site of the leakage during the study to treat the SRF. The participant’s visual acuity in the affected eye varied between 20/15 and 20/60 throughout her visits, depending on the extent of subretinal fluid present.

A 30-year-old healthy white female control subject was imaged during 22 research visits over 22 months. This participant received a complete eye exam prior to imaging studies and repeated AOSLO imaging at each visit and OCT imaging periodically throughout the 22 months. The participant was imaged with numerous protocols within our lab that encompassed large areas of the posterior pole as well as visits targeting more specific retinal areas [29,30,31,32]. At each visit, the control participant had 20/20 vision, no evidence of retinal pathology, and no systemic disease except for hypothyroidism. This study was approved by the Indiana University Institutional Review Board and adhered to the tenets of the Declaration of Helsinki. Informed consent was received from the participants.

### 2.2. Retinal Imaging

Prior to each imaging session, the choroidal nevus participant underwent a brief history, clinical exam, and was dilated with one drop each of 1% tropicamide ophthalmic solution and 2.5% phenylephrine hydrochloride ophthalmic solution. Imaging sessions captured large areas of the posterior pole with OCT and AOSLO, including the nevus and surrounding retina. During longitudinal imaging of the patient with the high-risk choroidal nevus we concentrated on two retinal regions: one with subretinal fluid and the other at the site of laser photocoagulation administered to treat SRF associated with the choroidal nevus.

#### 2.2.1. Optical Coherence Tomography

Volumetric spectral-domain optical coherence tomography (SD-OCT) scans (Spectralis, Heidelberg, Heidelberg, Germany) were acquired at each visit for the participant with the choroidal nevus. At a minimum, at least one 20° × 20° volumetric scan with a minimum of 48 b-scans (124 μm spacing) was obtained per session. Each scan encompassed the fovea, the choroidal nevus, and any areas of subretinal fluid (SRF) when present. Customized OCT scans were also performed as needed, varying in size and location depending on the extent of the serous detachment.

#### 2.2.2. Adaptive Optics Retinal Imaging

The custom dual-wavelength AOSLO system used in this study has been previously described [29,33]. AOSLO imaging was used to acquire simultaneous confocal and non-confocal or multiply scattered light images at various regions of interest (ROIs) over the duration of the study, using either a 1° × 1° or 2° × 2° field of view. Briefly, the confocal channel captures light directly backscattered from the imaged structure(s), while the non-confocal channel detects light that has been multiply scattered by structure(s) being imaged [23]. In the choroidal nevus participant, at every AOSLO imaging session, we captured a large region of the posterior pole concentrating on the borders between areas of attached retina and those detached by SRF. Imaging was performed at multiple depth planes for targeted ROIs, including the site of laser photocoagulation and regions above and below the SRF, when present. Although SRF often encompassed most of the macular region and extended into the arcades when present, beginning at the ninth visit through the twentieth visit, we also performed a through-focus series of a 2.1° × 1.4° ROI located approximately 2 degrees from the fovea, where the SRF fluctuated from visit to visit.

#### 2.2.3. Control Participant Imaging

In the control participant, imaging sessions captured large areas of the posterior pole, as described in previous studies [30,34], including regions captured in our participant with the choroidal nevus. The control participant also underwent more targeted imaging sessions where focal regions within the inner retina were obtained and imaged at repeated visits [31,32]. OCT volumes were captured periodically throughout the study period but not at every visit. AOSLO images of the cone photoreceptors and outer retina (8 visits) as well as vasculature and middle and inner retinal layers (14 visits) were obtained as well as OCT images. Only AOSLO imaging sessions with simultaneous confocal and multiply scattered light recordings were included in this analysis.

## 3. Results

We successfully navigated to distinct areas of interest in both our control and study participants over time. Unless otherwise noted, the following results refer to the study participant, as the control participant had normal retinal structure throughout the study.

### 3.1. Imaging with the Presence of SRF

During the imaging sessions when SRF was present, we made two unique observations. First, we were able to visualize in detail the RPE, which is not readily visible with AOSLO imaging in normal subjects. Second, we detected dynamic changes in hyper-reflective subretinal structures over time.

**Figure 2:**
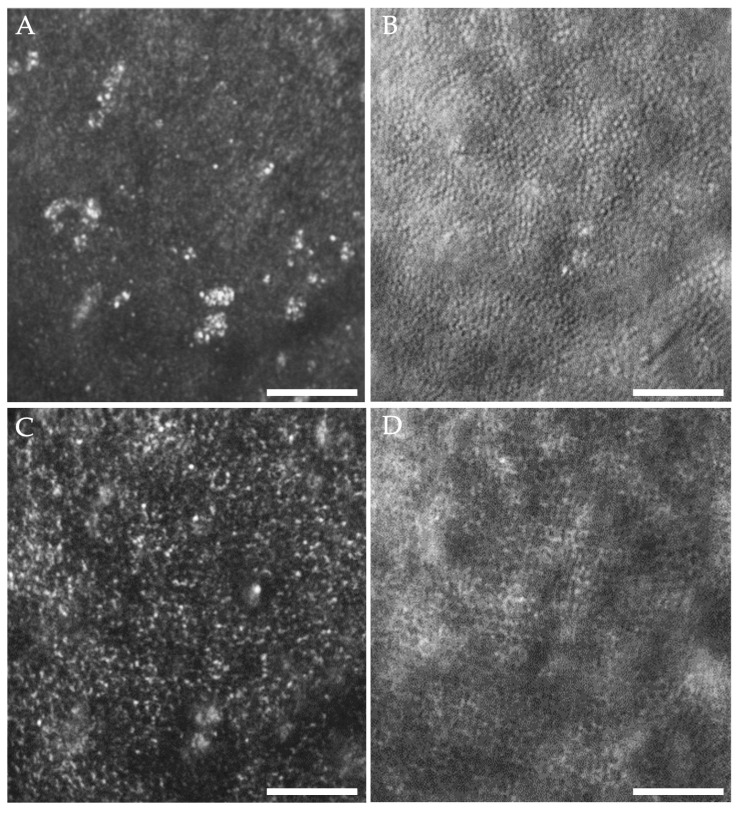
AOSLO images focused on photoreceptors (**A**,**B**) and RPE (**C**,**D**) in the same retinal location within an area of foveal subretinal fluid (SRF) due to the choroidal nevus. The pairs of (**A**,**B**) and (**C**,**D**) were each captured simultaneously using the confocal (**A**,**C**) and multiply scattered light channels (**B**,**D**). Note that in (**B**), the photoreceptor inner segment mosaic appears as expected, whereas only clusters of photoreceptors are visualized in (**A**) likely due to the presence of SRF altering the photoreceptors’ waveguiding ability. In both (**C**,**D**), the hexagonal RPE mosaic is seen in the presence of SRF. Image contrast was increased for visibility. A subset of the multiply scattered light data (**B**,**D**) has also been presented in Burns et al. [23]. Scale bars = 100 μm.

**Figure 3:**
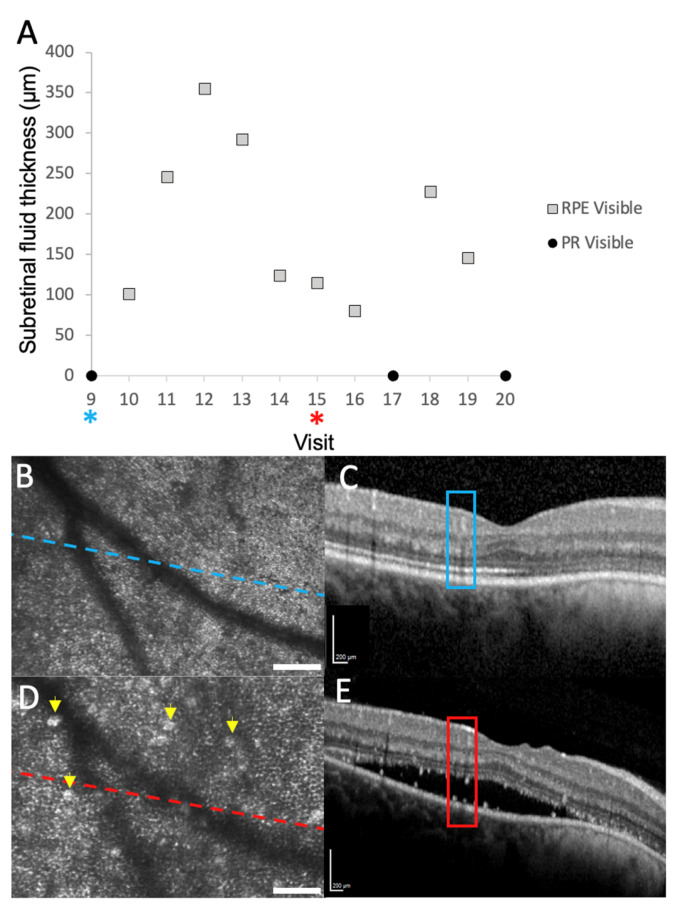
Plot of RPE visibility based on subretinal fluid (SRF) thickness 2 degrees temporal to the fovea. (**A**). Plot of visits, SRF thickness, and visibility of the RPE. In this ROI, the RPE was visible when any level of SRF was present (open square). When no SRF was present, only PRs could be visualized (black dot). (**B**). Confocal image of ROI showing intact PR mosaic in the absence of subretinal fluid. (**C**). SD-OCT cross section corresponding to (**B**). Blue square represents the area of (**B**) scan that corresponds to the dashed blue line in (**B**). (**B**,**C**) correspond to visit 9 (blue asterisk) in (**A**). There is no SRF present. (**D**). Confocal image focused at the level of the RPE demonstrates the hexagonal RPE mosaic. (**E**). SD-OCT cross-sections corresponding to (**D**). The red box represents that area of the b-scan that corresponds to the dashed red line in (**D**). (**D**,**E**) correspond to visit 15 in (**A**) (red asterisk). The thickness of the SRF at this visit was 394 μm. Numerous hyper-reflective foci are also visible in (**D**,**E**) (yellow arrows). Scale bars (**B**,**D**) = 100 μm.

When subretinal fluid was present, we could adjust the axial focus of our AOSLO to the plane of the displaced photoreceptors or through the SRF to the plane of the RPE. When focused on the photoreceptor layer, only clusters of visible cones can be seen in the confocal images (Figure 2A). However, multiply scattered light images revealed an intact inner segment mosaic (Figure 2B), suggesting that the presence of SRF may impair the waveguiding properties of the photoreceptor outer segments [35]. When we shifted focus to the RPE, the hexagonal array of RPE cells could be seen in both the confocal and multiply scattered light channels (Figure 2C,D). When no subretinal fluid was present (no displacement of photoreceptors), the RPE could no longer be seen despite adjustments in focus. In the 2.1° × 1.4° ROI that we began imaging at the ninth visit, the RPE mosaic remained visible whenever fluid was present, regardless of the depth of fluid (Figure 3A).

**Figure 4:**
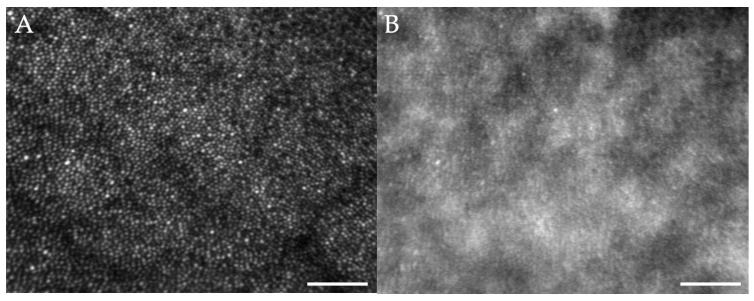
A 3 × 3-degree parafoveal region of the cone mosaic visualized with confocal (**A**) and non-confocal (**B**) light in the control subject. In (**B**), a spatial light modulator was used to capture the non-confocal image and the RPE hexagonal array can be visualized though much less distinctly than when subretinal fluid is present (compare Figure 2C,D). Scale bars = 50 µm.

Hyper-reflective foci were often observed within the areas of subretinal fluid with AOSLO and OCT imaging (Figure 3C,E) and may represent macrophages, microglia, migrating RPE cells, and/or lipid and proteinaceous material from damaged photoreceptors [36]. These structures changed markedly between visits. We were not able to co-localize them precisely between the two imaging modalities like what Vogel and colleagues observed when assessing similar structures in central serous chorioretinopathy [26]. In our control participant, whose retina was healthy and never had SRF, the RPE could not be visualized in 7 out of the 8 imaging sessions that included cones. However, in one parafoveal region, some of the hexagonal arrangement of the RPE could be visualized in the non-confocal image (Figure 4) generated with a configurable aperture [29].

### 3.2. Imaging at the Site of Laser Photocoagulation

After the first imaging visit, the patient underwent focal laser photocoagulation by her retinal oncologist to treat the SRF induced by the choroidal nevus. We identified the location of laser photocoagulation using her fluorescein angiogram obtained prior to the procedure.

**Figure 5:**
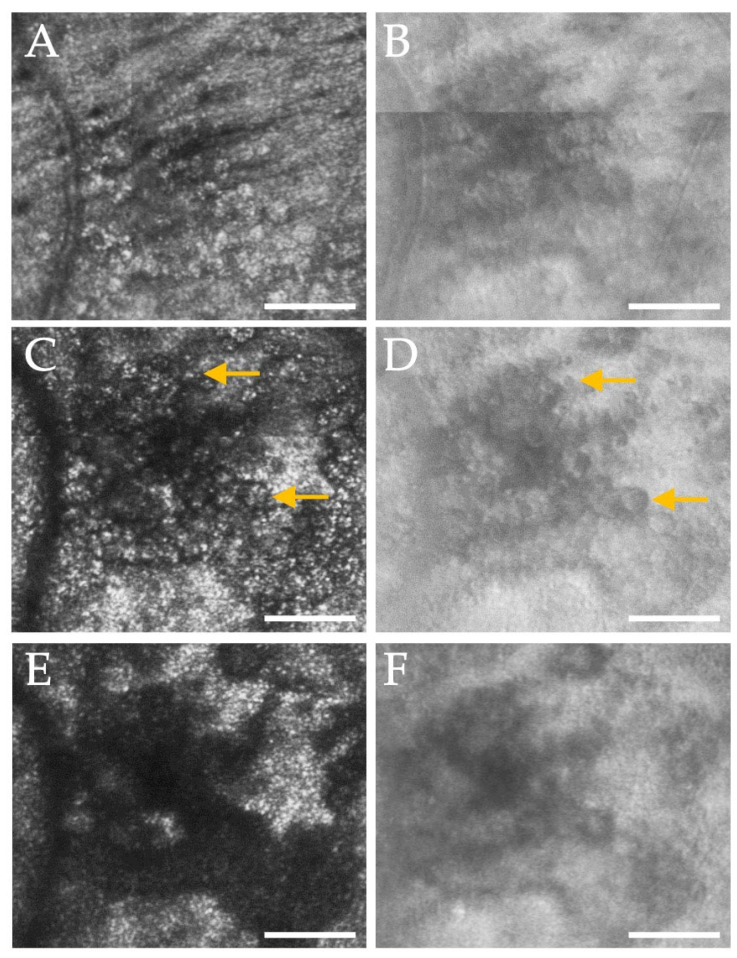
Cellular infiltrates at site of laser photocoagulation 20 days post treatment. Confocal (**A**,**C**,**E**) and multiply scattered light (**B**,**D**,**F**) images focused on the nerve fiber layer (**A**,**B**), just above the photoreceptors (**C**,**D**), and at the photoreceptors (**E**,**F**). Cellular infiltrates are just above the photoreceptors and examples of a small and large cell-like structure are shown with the yellow arrows. Contrast adjusted for visibility. Scale bars = 100 µm.

The site of laser photocoagulation was approximately 375 H × 423 V µm. Twenty days after the laser photocoagulation procedure was performed, the patient returned for AOSLO imaging. A through-focus series was performed at the site of laser photocoagulation (Figure 5). Multiple distinct, cell-like structures infiltrated the middle and outer retina (Figure 5C) and were observed most evidently just above the photoreceptors. These circular structures had clearly delineated walls, visualized in the multiply scattered light AOSLO images, and often contained numerous granules of hyper-reflective material, best visualized in the confocal images. Sixty-six of these cell-like structures could be clearly counted and visualized, ranging in size from 10 µm to 39 µm, with an average size of 20 µm. When focused on the level of the photoreceptors, approximately 1/3 of the cones in this region appeared bright, presumably due to light guiding, and the remainder appeared dim (Figure 5E). At 34 days post laser, the shape of this region had changed slightly and decreased to approximately 311 by 249 µm. Several cell-like bodies were still visible, 52 of which could be counted and measured and ranged in size between 6 and 26 µm, with an average size of 17 µm. These cell-like structures are presumed to be macrophages and microglia, which are known to infiltrate areas after retinal laser photocoagulation [37,38]. At 158 days post laser, the borders of this region could no longer be distinctly visualized, and only a few cell-like structures could be seen in the multiply scattered light images. The photoreceptor layer had returned to a more normal appearance with waveguiding cones visualized over the entire region of treatment. All regions were compared with SD-OCT and, while clear disruption and larger cavitations were visualized in the outer retina, there was no cystic edema corresponding in size to the circular structures visualized with AOSLO.

**Figure 6:**
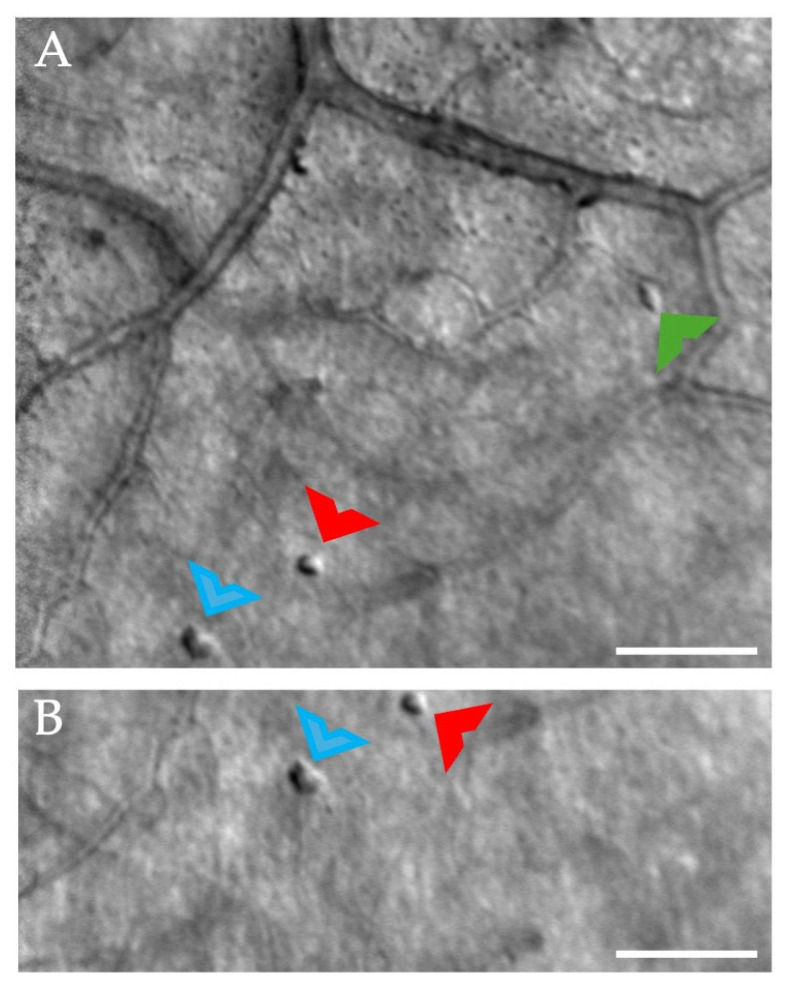
Example of cell-like structures over time in the control participant, superior nasal to the foveal avascular zone border. (**A**). Arrowheads (red, blue, and green) point to cell-like structures. (**B**). Two of the cell-like structures (red and blue arrowheads) are still visible at the same location one month later, exhibiting the same size and morphology. Scale bars = 50 µm.

In our control participant, few cell-like structures were visible at times sporadically throughout the imaging sessions. These structures were often isolated, not clustered, and appeared in various locations throughout the posterior pole. No retinal abnormalities were detected in the locations in which these cell-like structures were visible. Unlike our study participant, these structures remained stable over the course of one month (Figure 6), and their appearance is similar to presumed microglia previously reported with AOSLO imaging [39].

## 4. Discussion

In this study, we performed longitudinal, high-resolution retinal imaging in a study participant with a choroidal nevus and subretinal fluid who underwent therapeutic laser photocoagulation. We were able to repeatedly capture AOSLO images of large regions of the retina and easily navigate to small ROIs in both our study and control participant. The use of the steering mirrors [33,40] and a navigation module [41] for the AOSLO systems used in this study enabled us to efficiently locate the two regions even when the participant’s visual acuity and image quality were impacted by the presence of SRF. It also enabled us to return to smaller ROIs repeatedly with each consecutive imaging session. Thus, we were able to visualize and measure the dynamic nature of the retinal response to the disease and laser treatment over time. This longitudinal imaging approach can be generalized to other retinal pathologies, and our primary findings include that the RPE cells can be reliably imaged through SRF and that infiltration of macrophage-like cells is visualized in the retinal response to laser photocoagulation.

### 4.1. RPE Visualization

Subretinal fluid (SRF) or serous neurosensory retinal detachments may occur in numerous retinal disease pathologies, including diabetic macular edema, central serous chorioretinopathy, and macular degeneration. With AOSLO imaging, we demonstrate that we can consistently focus our image plane through the fluid and image the RPE mosaic when subretinal fluid was present, regardless of the depth of the fluid (Figure 4A). In normal participants, the RPE is difficult to visualize with AOSLO due to the proximity of the photoreceptors that generate strong backscatter of the imaging light from the instrument. Reliably visualizing the RPE mosaic in healthy subjects with AOSLO would require the use of a systemic dye [42] or autofluorescence capabilities [43]. Blocking the confocal signal to various degrees can also allow some visualization of the RPE cells [44] but does not enable reliable RPE imaging across numerous subjects in our experience. In our control subject, we were able to poorly visualize RPE in one imaging session (Figure 5B). However, this was performed using a spatial light modulator (SLM) and reconfiguring the aperture pattern on the SLM [29], requiring operator expertise and being time-consuming. When photoreceptors are damaged or lost, the RPE mosaic may also be visualized [45]. Like this study, Scoles and colleagues [44] also demonstrated the ability to image the RPE when the photoreceptors are displaced due to subretinal fluid in CSC. Furthermore, the signal from individual photoreceptors is still apparent in the non-confocal image of the RPE in our control participant (Figure 5B), whereas it is nearly absent in our study participant (Figure 2D) and with the use of an offset aperture. This suggests an opportunity to study photoceptor recovery and RPE alterations in other conditions with neurosensory retinal detachments. AO-OCT, which has improved axial resolution in comparison to AOSLO systems, reliably enables the visualization of RPE in normal humans [46].

### 4.2. Hyper-Reflective Foci and Clusters

The dynamic nature of hyper-reflective foci was evident, and clusters were present within and at the borders of the subretinal fluid. These foci could be visualized with AOSLO and OCT imaging, although they were difficult to co-localize. These structures varied in size and location from visit to visit. Vogel and colleagues [26] reported similar findings in CSC, where some of the clusters could be co-localized between both AOSLO and OCT. In this case study, we were unable to correlate AOSLO and OCT findings consistently due to variations in the OCT scan protocol on visits, but our findings were similar to the type-1 clusters in CSC [26] and likely represent migration of microglia, macrophages, protein or lipid material, and migratory RPE cells in response to the neurosensory retinal detachment in this participant [26,36,47]. While the significance of these findings is still under debate, we show that these foci and clusters can be followed over time in response to retinal disease. Similar structures have been described in numerous pathologies with and without neurosensory retinal detachments and hyper-reflective foci may serve as a predictive biomarker for progression in diabetic retinopathy and age-related macular degeneration [36].

### 4.3. Site of Laser Photocoagulation

The response to laser photocoagulation was also dynamic, with cell-like structures infiltrating the treatment site, followed by remodeling. In a rat model, strong glial and inflammatory cell responses were observed at the site of laser photocoagulation with an Nd:Yag laser [48]. The response included an initial response by microglia and a later infiltration by macrophages from 3 to 7 days post treatment, which was the extent of longitudinal monitoring [48]. In rabbits, migrating RPE cells have also been found to infiltrate an area of laser photocoagulation, and these cells disappeared within 4 months post laser [49]. Recently, Power and colleagues [50] have demonstrated a strong microglial response to retinal laser photocoagulation observed in vivo with AOSLO and ex vivo in a mouse model. We observed cellular infiltrates, presumably immune cells such as macrophages or microglia, twenty days post laser (our first measurement point), and these cells gradually dissipated over time. The confocal images of these cells (Figure 5C) demonstrated that there were often several hyperreflective granules within the circular structure visualized on the non-confocal image (Figure 5D) which could represent phagosomes within these cell-like structures [51]. While our participant with the choroidal nevus underwent therapeutic laser photocoagulation, imaging of additional individuals undergoing such treatment for various retinal conditions may help us better understand the retinal immune response in humans.

Laser injury and photocoagulation can also cause disorganization and disruption to the photoreceptor outer segments [48,49]. Confocal AOSLO images in this participant showed a large, hypo reflective region at the site of laser photocoagulation with no cone reflectance visible (Figure 4). We presume that our participant’s outer segments were disrupted in a manner like that observed in both animal models and human studies involving laser photocoagulation [27]. Wang et al. showed similar findings but without recovery after a laser injury [28]. In our participant, the outer segment reflectivity gradually increased over time, indicating some recovery of the outer segments, and Wang et al. has demonstrated that retinal sensitivity at the site of laser injury, which is presumably higher level of energy exposure than in our patient, returned to normal even with persistence of a defect in the cone mosaic [28].

At times, cell-like structures were visualized in our control participant (Figure 6). When compared to our study participant, these structures remained relatively stable over time and were located sporadically throughout the retina, and retinal structures, mostly blood vessels, adjacent to these cells, were all normal in appearance. Rui and colleagues [39] recently demonstrated imaging of putative microglia in healthy control participants and an upregulation of these cells in active uveitis. The structures identified in our control participant are similar in appearance, and thus, could also represent presumed microglial cells.

### 4.4. Limitations

While OCTs were collected at each visit, the scan size and density varied due to the dynamic nature of the fluid elevation, making it difficult to directly correlate AOSLO findings with those on clinical OCT. The participant did not return for research imaging until twenty days after the procedure, limiting our ability to measure the earliest phase of the retinal response. However, as these injuries persisted for months, our findings still represent measurements of the presumed retinal immune response to injury in a living human using AOSLO imaging. Lastly, we only have one subject with retinal sequalae due to a choroidal nevus, which limits the generalizability of this study. However, we do show the opportunity to further monitor such alterations longitudinally. As laser photocoagulation may serve to study immune response in animal models [50], monitoring humans with high resolution retinal imaging who have undergone therapeutic laser photocoagulation may provide an insight into the immune response as well.

## 5. Conclusions

Longitudinal AOSLO and OCT measurements can be used to monitor dynamic changes in the retina, documenting both the natural history and the response to treatment. We were able to image large areas of the retina as well as repeatedly return to smaller ROIs presented here at subsequent visits, which allowed us to use these imaging modalities to follow the time course of disease progression. We could clearly visualize the RPE when SRF was present. The presence of SRF and laser photocoagulation induce dynamic changes that occur in response to retinal disruption, including the infiltration of cell-like structures. These structures are presumed to be macrophage-like cells responding to the disruption induced by SRF and laser photocoagulation. Our findings demonstrate the value of longitudinal imaging and the opportunity to monitor the inflammatory response in the outer retina due to both pathological and therapeutic insults. Further investigations into these structures with AOSLO and OCT will provide an insight into their utility as a biomarker for disease progression and retinal inflammation.

## Data Availability

Data underlying the results presented in this paper are not publicly available at this time but may be obtained from the authors upon reasonable request.

## Abbreviations

The following abbreviations are used in this manuscript:

PR	Photoreceptor
TFSOM-UHH	To find small ocular melanoma using helpful hints
OCT	Optical coherence tomography
OCTA	Optical coherence tomography angiography
SD-OCT	Spectral-domain optical coherence tomography
AOSLO	Adaptive optics scanning laser ophthalmoscopy
SRF	Subretinal fluid
RPE	Retinal pigment epithelium
CSC	Central serous chorioretinopathy
ROI	Region of interest
FAF	Fundus autofluorescence

## References

[1] Singh A.D., Kalyani P., Topham A. (2005). Estimating the risk of malignant transformation of a choroidal nevus. Ophthalmology.

[2] Greenstein M.B., Myers C.E., Meuer S.M., Klein B.E., Cotch M.F., Wong T.Y., Klein R. (2011). Prevalence and characteristics of choroidal nevi: The multi-ethnic study of atherosclerosis. Ophthalmology.

[3] Jonas J.B., You Q.S., Xu L., Wang Y.X. (2008). Choroidal Nevi in Adult Chinese. Ophthalmology.

[4] Ng C.H., Wang J.J., Mitchell P., Amirul Islam F.M., Wong T.Y. (2009). Prevalence and Characteristics of Choroidal Nevi in an Asian vs White Population. Arch. Ophthalmol..

[5] Shields C.L., Furuta M., Berman E.L., Zahler J.D., Hoberman D.M., Dinh D.H., Mashayekhi A., Shields J.A. (2009). Choroidal nevus transformation into melanoma: Analysis of 2514 consecutive cases. Arch. Ophthalmol..

[6] Shields C.L., Demirci H., Materin M.A., Marr B.P., Mashayekhi A., Shields J.A. (2004). Clinical factors in the identification of small choroidal melanoma. Can. J. Ophthalmol..

[7] Sallet G., Amoaku W.M.K., Lafaut B.A., Brabant P., De Lacy J.J. (1995). Indocyanine green angiography of choroidal tumors. Graefe’s Arch. Clin. Exp. Ophthalmol..

[8] Shields C.L., Dalvin L.A., Ancona-Lezama D., Yu M.D., Di Nicola M., Williams B.K., Lucio-Alvarez J.A., Ang S.M., Maloney S., Welch R.J. (2019). Choroidal nevus imaging features in 3806 cases and risk factors for transformation into melanoma in 2355 cases: The 2020 Taylor R. Smith and Victor T. Curtin Lecture. Retina.

[9] Chien J.L., Sioufi K., Surakiatchanukul T., Shields J.A., Shields C.L. (2017). Choroidal nevus: A review of prevalence, features, genetics, risks, and outcomes. Curr. Opin. Ophthalmol..

[10] Verbeek S., Dalvin L.A. (2024). Advances in multimodal imaging for diagnosis of pigmented ocular fundus lesions. Can. J. Ophthalmol..

[11] Yu M.D., Heiferman M., Korot E., Ahluwalia A., Yu G., Mruthyunjaya P. (2025). Pixel Intensity to Estimate Choroidal Tumor Thickness Using 2-Dimensional Ultra-Widefield Images. JAMA Ophthalmol..

[12] Damato B.E. (2023). Can the MOLES acronym and scoring system improve the management of patients with melanocytic choroidal tumours?. Eye.

[13] Damato B.E., Foulds W.S. (1990). Tumour-associated retinal pigment epitheliopathy. Eye.

[14] Naylor A., Hopkins A., Hudson N., Campbell M. (2019). Tight Junctions of the Outer Blood Retina Barrier. Int. J. Mol. Sci..

[15] Gonder J.R., Augsburger J.J., McCarthy E.F., Shields J.A. (1982). Visual loss associated with choroidal nevi. Ophthalmology.

[16] Shields C.L., Furuta M., Mashayekhi A., Berman E.L., Zahler J.D., Hoberman D.M., Dinh D.H., Shields J.A. (2007). Visual Acuity in 3422 Consecutive Eyes With Choroidal Nevus. Arch. Ophthalmol..

[17] Goldman D.R., Barnes A.C., Vora R.A., Duker J.S. (2014). Leaking choroidal nevus treated with focal laser photocoagulation. Retin. Cases Brief Rep..

[18] Querques G., Prascina F., Iaculli C. (2008). Focal laser photocoagulation for polypoidal choroidal vasculopathy associated with choroidal nevus. Retin. Cases Brief Rep..

[19] Maltsev D.S., Kulikov A.N., Burnasheva M.A., Chhablani J. (2023). Photoreceptor outer segment layer thinning as a biomarker in acute central serous chorioretinopathy. Ther. Adv. Ophthalmol..

[20] Yoon J., Han J., Ko J., Choi S., Park J.I., Hwang J.S., Han J.M., Jang K., Sohn J., Park K.H. (2022). Classifying central serous chorioretinopathy subtypes with a deep neural network using optical coherence tomography images: A cross-sectional study. Sci. Rep..

[21] Baek J., Cheung C.M.G., Jeon S., Lee J.H., Lee W.K. (2019). Polypoidal Choroidal Vasculopathy: Outer Retinal and Choroidal Changes and Neovascularization Development in the Fellow Eye. Investig. Opthalmol. Vis. Sci..

[22] Khan K.N., Mahroo O.A., Khan R.S., Mohamed M.D., McKibbin M., Bird A., Michaelides M., Tufail A., Moore A.T. (2016). Differentiating drusen: Drusen and drusen-like appearances associated with ageing, age-related macular degeneration, inherited eye disease and other pathological processes. Prog. Retin. Eye Res..

[23] Burns S.A., Elsner A.E., Sapoznik K.A., Warner R.L., Gast T.J. (2019). Adaptive optics imaging of the human retina. Prog. Retin. Eye Res..

[24] Rodrigues M.W., Correa Z.M., Say E.A., Borges F.P., Siqueira R.C., Cardillo J.A., Jorge R. (2016). Photoreceptor Arrangement Changes Secondary to Choroidal Nevus. JAMA Ophthalmol..

[25] Rodrigues M.W., Say E.A., Shields C.L., Jorge R. (2017). Adaptive Optics of Small Choroidal Melanoma. Ophthalmic Surg. Lasers Imaging Retin..

[26] Vogel R.N., Langlo C.S., Scoles D., Carroll J., Weinberg D.V., Kim J.E. (2017). High-Resolution Imaging of Intraretinal Structures in Active and Resolved Central Serous Chorioretinopathy. Investig. Ophthalmol. Vis. Sci..

[27] Wood E.H., Leng T., Schachar I.H., Karth P.A. (2016). Multi-Modal Longitudinal Evaluation of Subthreshold Laser Lesions in Human Retina, Including Scanning Laser Ophthalmoscope-Adaptive Optics Imaging. Ophthalmic Surg. Lasers Imaging Retin..

[28] Wang Y., La T.T., Mason M., Tuten W.S., Roorda A. (2023). Case Report: Multimodal, Longitudinal Assessment of Retinal Structure and Function following Laser Retinal Injury. Optom. Vis. Sci..

[29] Luo T., de Castro A., Sawides L., Warner R.L., Burns S.A. (2018). Enhanced retinal vasculature imaging with a rapidly configurable aperture. Biomed. Opt. Express.

[30] Sapoznik K.A., Gast T.J., Carmichael-Martins A., Walker B.R., Warner R.L., Burns S.A. (2023). Retinal Arteriolar Wall Remodeling in Diabetes Captured With AOSLO. Transl. Vis. Sci. Technol..

[31] Warner R.L., de Castro A., Sawides L., Gast T., Sapoznik K., Luo T., Burns S.A. (2020). Full-field flicker evoked changes in parafoveal retinal blood flow. Sci. Rep..

[32] Warner R.L., Gast T.J., Sapoznik K.A., Carmichael-Martins A., Burns S.A. (2021). Measuring Temporal and Spatial Variability of Red Blood Cell Velocity in Human Retinal Vessels. Investig. Ophthalmol. Vis. Sci..

[33] Ferguson R.D., Zhong Z., Hammer D.X., Mujat M., Patel A.H., Deng C., Zou W., Burns S.A. (2010). Adaptive optics scanning laser ophthalmoscope with integrated wide-field retinal imaging and tracking. J. Opt. Soc. Am. A Opt. Image Sci. Vis..

[34] Sawides L., Sapoznik K.A., de Castro A., Walker B.R., Gast T.J., Elsner A.E., Burns S.A. (2017). Alterations to the Foveal Cone Mosaic of Diabetic Patients. Investig. Ophthalmol. Vis. Sci..

[35] Scoles D., Sulai Y.N., Langlo C.S., Fishman G.A., Curcio C.A., Carroll J., Dubra A. (2014). In vivo imaging of human cone photoreceptor inner segments. Investig. Ophthalmol. Vis. Sci..

[36] Fragiotta S., Abdolrahimzadeh S., Dolz-Marco R., Sakurada Y., Gal-Or O., Scuderi G. (2021). Significance of Hyperreflective Foci as an Optical Coherence Tomography Biomarker in Retinal Diseases: Characterization and Clinical Implications. J. Ophthalmol..

[37] Conedera F.M., Kokona D., Zinkernagel M.S., Stein J.V., Lin C.P., Alt C., Enzmann V. (2024). Macrophages coordinate immune response to laser-induced injury via extracellular traps. J. Neuroinflamm..

[38] Eter N., Engel D.R., Meyer L., Helb H.-M., Roth F., Maurer J., Holz F.G., Kurts C. (2008). In Vivo Visualization of Dendritic Cells, Macrophages, and Microglial Cells Responding to Laser-Induced Damage in the Fundus of the Eye. Investig. Ophthalmol. Vis. Sci..

[39] Rui Y., Zhang M., Lee D.M.W., Snyder V.C., Raghuraman R., Gofas-Salas E., Mecê P., Yadav S., Tiruveedhula P., Grieve K. (2024). Label-Free Imaging of Inflammation at the Level of Single Cells in the Living Human Eye. Ophthalmol. Sci..

[40] Burns S.A., Tumbar R., Elsner A.E., Ferguson D., Hammer D.X. (2007). Large-field-of-view, modular, stabilized, adaptive-optics-based scanning laser ophthalmoscope. J. Opt. Soc. Am. A Opt. Image Sci. Vis..

[41] Huang G., Qi X., Chui T.Y.P., Zhong Z., Burns S.A. (2012). A Clinical Planning Module for Adaptive Optics SLO Imaging. Optom. Vis. Sci..

[42] Tam J., Liu J., Dubra A., Fariss R. (2016). In Vivo Imaging of the Human Retinal Pigment Epithelial Mosaic Using Adaptive Optics Enhanced Indocyanine Green Ophthalmoscopy. Investig. Ophthalmol. Vis. Sci..

[43] Morgan J.I., Dubra A., Wolfe R., Merigan W.H., Williams D.R. (2009). In vivo autofluorescence imaging of the human and macaque retinal pigment epithelial cell mosaic. Investig. Ophthalmol. Vis. Sci..

[44] Scoles D., Sulai Y.N., Dubra A. (2013). In vivo dark-field imaging of the retinal pigment epithelium cell mosaic. Biomed. Opt. Express.

[45] Roorda A., Zhang Y., Duncan J.L. (2007). High-Resolution In Vivo Imaging of the RPE Mosaic in Eyes with Retinal Disease. Investig. Ophthalmol. Vis. Sci..

[46] Liu Z., Kocaoglu O.P., Miller D.T. (2016). 3D Imaging of Retinal Pigment Epithelial Cells in the Living Human Retina. Investig. Ophthalmol. Vis. Sci..

[47] Lewis G.P., Sethi C.S., Carter K.M., Charteris D.G., Fisher S.K. (2005). Microglial cell activation following retinal detachment: A comparison between species. Mol. Vis..

[48] Chidlow G., Shibeeb O.S., Plunkett M., Casson R.J., Wood J.P.M. (2013). Glial Cell and Inflammatory Responses to Retinal Laser Treatment: Comparison of a Conventional Photocoagulator and a Novel, 3-Nanosecond Pulse Laser. Investig. Ophthalmol. Vis. Sci..

[49] Paulus Y.M., Jain A., Gariano R.F., Stanzel B.V., Marmor M., Blumenkranz M.S., Palanker D. (2008). Healing of Retinal Photocoagulation Lesions. Investig. Ophthalmol. Vis. Sci..

[50] Power D., Elstrott J., Schallek J. (2025). Photoreceptor loss does not recruit neutrophils despite strong microglial activation. eLife.

[51] Lückoff A., Scholz R., Sennlaub F., Xu H., Langmann T. (2017). Comprehensive analysis of mouse retinal mononuclear phagocytes. Nat. Protoc..

